# A pilot study comparing virtual treatment setups among clear aligner companies

**DOI:** 10.1590/2177-6709.30.3.e2524243.oar

**Published:** 2025-10-20

**Authors:** Vicente TELLES, Claudia Trindade MATTOS, Flavio COPELLO, Maria B. DOUGHAN

**Affiliations:** 1 University of Maryland Baltimore, Dental School, Department of Orthodontics (Baltimore, USA).; 2 Fluminense Federal University, Dental School, Department of Orthodontics (Niterói/RJ, Brazil).; 3 University of Maryland Baltimore, Dental School, Department of Orthodontics and Pediatric Dentistry (Baltimore, USA).

**Keywords:** Clear aligner appliances, Orthodontic appliance designs, Clinical decision-making, Alinhadores, Design de aparelhos ortodônticos, Decisão clínica

## Abstract

**Objective:**

This pilot study aims to compare virtual treatment setups provided by four different clear aligner companies, to assess how variations in features might impact treatment planning.

**Methods:**

Initial records of 10 patients were submitted to Invisalign®, ClearCorrect®, 3M™ Clarity™, and Spark™. Standardized case prescriptions ensured comparable treatment plans across companies. Comparisons focused on the number of aligners, number of attachments, interproximal reduction per arch, vertical movement of maxillary central incisors, final canine and molar relationships, intercanine and intermolar widths, and expansion or constriction of these widths.

**Results:**

Among the companies, significant differences were found in the number of aligners (p = 0.003), number of attachments (p< 0.001), and final canine relationship (p = 0.013). No statistical differences were observed for the other variables. ClearCorrect® prescribed the fewest number of aligners and attachments, while 3M™ Clarity™ prescribed the most. ClearCorrect® and Spark™ showed deficiencies in planning for bilateral canine Class I relationship.

**Conclusions:**

The unique characteristics of each aligner company result in distinct approaches to treating the same patient, highlighting both areas of discrepancy and consistency in virtual treatment setups.

## RESUMO

**Objetivo:**

Este estudo piloto tem como objetivo comparar o *setup* virtual entre quatro empresas de alinhadores para avaliar como as características de cada uma podem impactar no plano de tratamento.

**Métodos:**

A documentação inicial de 10 pacientes foi enviada para Invisalign^®^, ClearCorrect^®^, 3M™ Clarity™, e Spark™. A prescrição de tratamento para cada caso foi padronizada para garantir planos de tratamento equiparáveis entre as empresas. As comparações focaram no número de alinhadores, número de *attachments*, redução interproximal por arco, movimento vertical dos incisivos centrais superiores, relações oclusais finais de caninos e molares, distâncias intercaninos e intermolar, e expansão ou constrição dessas distâncias.

**Resultados:**

Entre as empresas, diferenças significativas foram encontradas no número de alinhadores (p-valor = 0,003), número de *attachments* (p-valor < 0,001) e relação oclusal final dos caninos (p-valor = 0,013). Não foram observadas diferenças estatísticas para as outras variáveis. A ClearCorrect^®^ prescreveu o menor número de alinhadores e *attachments*, enquanto a 3M™ Clarity™ prescreveu o maior. A ClearCorrect^®^ e a Spark™ apresentaram deficiências no planejamento para obtenção de relação bilateral de Classe I dos caninos.

**Conclusões:**

As características individuais de cada empresa de alinhadores resultam em abordagens distintas para o tratamento de um mesmo paciente, destacando-se tanto áreas de discrepância quanto de consistência nas configurações do *setup* virtual.

## INTRODUCTION

Over the past two decades, clear aligner therapy has emerged as an alternative to fixed brackets in orthodontic treatment. A 2022 survey revealed that younger orthodontists believe clear aligners will become the primary technique for treating malocclusions in the future.^1^ Invisalign^®^ led the market for 20 years, but after their patents expired in 2017, other companies began developing their own aligner brands, fostering competition and innovation.^2^ ^-^ ^6^ These companies vary in material type and thickness, gingival trim design, auxiliary tools, and orthodontic movement strategies.^4^ This diversity has shifted the perception of clear aligners from a single system to multiple distinct systems.^7^

A crucial component of clear aligner therapy is the virtual treatment setup (VTS), which allows for precise planning and customization of the aligners to meet each patient’s needs. Despite each company’s unique characteristics, little is known about their impact on treatment setup. According to Putrino et al,^7^ 90% of research publications on clear aligners center exclusively on Invisalign^®^, leaving a knowledge gap regarding other brands. 

This study aimed to understand how the features of four different aligner brands (Invisalign^®^, ClearCorrect^®^, 3M™ Clarity™ Aligners, and Spark™) affect their VTS. To achieve this, we compared the VTS for the same ten patients across these companies. The null hypothesis was that all companies would provide similar VTS for the same patient, while the alternative hypothesis proposed that the VTS would differ among the companies.

## MATERIAL AND METHODS

The University of Maryland Institutional Review Board approved the study under protocol #HP-00102571, on November 2^nd^, 2022. All procedures were performed in compliance with relevant laws and institutional guidelines.

This pilot study included a convenience sample of 10 patients who met the following inclusion criteria: (1) availability of pretreatment records, including digital models and intra and extraoral photographs; (2) ages between 12 and 40 years; (3) presence of all permanent teeth except for third molars; and (4) overall healthy status. The exclusion criteria were: (1) patients requiring orthognathic surgery or those with syndromes; (2) individuals with fixed prostheses such as implants or crowns; and (3) those in deciduous or mixed dentition. All patients were undergoing fixed orthodontic treatment and were informed that their initial records would be used solely to generate VTS by different clear aligner companies, without impacting their ongoing treatments. Informed consent and research assent were obtained from all patients and their guardians, when applicable.

The clear aligner companies selected for this study included Invisalign^®^, ClearCorrect^®^, 3M™ Clarity™ Aligners, and Spark™. The various characteristics of the aligners provided by each company are presented in Table 1.^8^ ^-^ ^12^ Before submitting the records, several preemptive measures were implemented, to reduce confounding factors. First, clinical preferences for each aligner company were standardized to ensure the best compatibility across all companies. These overarching preferences are applied by default to every case submitted to the aligner company and encompass specific details, such as preferred size and timing of attachment placement, staging for interproximal reduction (IPR), preferred tooth numbering system, maximum accepted arch expansion per quadrant, preferred final overjet and overbite measurements, and an aligner change protocol set to 14 days, following the default setting recommended by all included aligner companies. Second, the prescription for each case and aligner company was reviewed by two investigators (V.T. and M.D.) to confirm an optimal match. In instances of disagreement, these investigators engaged in discussions to determine the most suitable approach. All prescriptions were selected through multiple-choice options and/or drop-down menus, and no written special instructions were entered to minimize the input of the laboratory technicians and increase reliance on the artificial intelligence and machine learning algorithms inherent to each company’s software. A summary of each case prescription is presented on Table 2. 

**Table 1:** Comparison of aligner characteristics across companies.

Aligner Brand	Company	Release date	Planning software	Type of plastic	Description of plastic	Aligner design		
						Thickness (mm)	Trim design	Gingival trim height
Invisalign^8^ ^,^ ^9^	Align Technology	1998	ClinCheck	SmartTrack	Multi-layer aromatic thermoplastic polyurethane	0.75	Scalloped	At the gingival margin
ClearCorrect^10^	Straumann	2009	ClearPilot	ClearQuartz	Tri-layer: elastomeric inner layer between two resilient, low-porosity polymers	0.75	Flat	2 mm above the gingival margin
Clarity^11^	3M	2018	3M Tx Designer	Flex	Five-layer blend for flexibility	0.625	Flat	At the gingival margin
				Force	Conventional rigid plastic	0.75		
Spark^12^	Ormco™	2019	Approver	TruGen	Greater flexibility	0.75	Scalloped	At the gingival margin
				TruGen XR	Increased rigidity	0.75		

**Table 2:** Characteristics of patients’ malocclusion and summary of case prescriptions.

Patient	Malocclusion	Case prescription
14-year-old, female	Bilateral molar Class III and canine Class I relationship, bimaxillary protrusion, mild maxillary and mandibular spacing, and anterior open bite (2 mm)	Four first premolar extractions to address bimaxillary protrusion and improve open bite by relative extrusion of anterior teeth during retraction.
15-year-old, male	Bilateral molar Class I and canine Class II relationship, severe maxillary and mandibular crowding, deep overbite.	Four first premolar extractions to address crowding and correction of deep overbite by intrusion of lower anterior teeth and use of bite ramps.
14-year-old, male	Bilateral molar Class I relationship, right canine Class I and left canine Class II relationship, bimaxillary protrusion, with moderate spacing on the maxillary and mandibular arches, and anterior open bite (5 mm)	Close the spaces by retraction of the incisors, with extrusion of the maxillary anterior teeth to close the anterior open bite.
21-year-old, female	Bilateral molar and canine Class I relationship, bimaxillary protrusion, with severe maxillary and mandibular spacing.	Close the spaces by retracting the anterior teeth.
13-year-old, male	Bilateral molar and canine Class II relationship, retroclined maxillary and mandibular incisors, with deep overbite, and mild maxillary and mandibular crowding.	Use of Class II elastics, correction of deep bite by intrusion of lower anterior teeth and use of bite ramps, and proclinatiton of maxillary and mandibular incisors to relieve crowding, with expansion and IPR as needed.
17-year-old, female	Bilateral molar and canine Class I relationship, with left canine in crossbite, mild spacing on maxillary and mandibular arches.	Close the spaces, addressing the crossbite.
14-year-old, female	Bilateral molar Class I and canine Class II relationship, with mild crowding on the maxillary and mandibular arches, and increased overjet.	Preference for IPR to address the crowding, without expansion or proclination.
14-year-old, female	Bilateral molar and canine Class II relationship, with severe maxillary and moderate mandibular crowding, and increased overjet.	Extraction of maxillary first premolars and mandibular second premolars, use of Class II elastics.
14-year-old, female	Bilateral molar and canine Class I relationship, with mild maxillary spacing and mild mandibular crowding.	Close the spaces and resolve the crowding.
15-year-old, female	Bilateral molar and canine Class I relationship, with mild maxillary spacing and moderate mandibular crowding, retroclined upper and lower incisors, and deep overbite.	Use of bite ramps, Class II elastics, and intrusion of mandibular incisors to address deep overbite, with proclination and expansion of the incisors as the primary movement, planning IPR as needed.

To ensure a comprehensive comparison of the various virtual treatment setups, the selected variables included:

1. The prescribed number of aligners,
2. The prescribed number of attachments,
3. The prescribed amount of interproximal reduction (IPR) for the maxillary arch,
4. The prescribed amount of IPR for the mandibular arch,
5. The planned extrusive or intrusive movement of maxillary central incisors,
6. The predicted final canine relationship,
7. The predicted final molar relationship,
8. The predicted final mandibular intercanine width,
9. The planned expansion or constriction of the mandibular intercanine width,
10. The predicted final mandibular intermolar width, and
11. The planned expansion or constriction of the mandibular intermolar width.

Attachments were counted per tooth, even if two were placed on the same tooth. The extrusion/intrusion values of maxillary central incisors were averaged from both sides and obtained from the tooth movement table, with positive values indicating extrusion and negative values indicating intrusion.

The final canine and molar relationships were assessed, scoring a “Yes” for bilateral Class I and a “No” otherwise. The canine classification was based on the anteroposterior relationship of the maxillary canine cusp tip and the interproximal contact between the mandibular canine and the first premolar. Molar classification was based on the American Board of Orthodontics standards.^13^

The mandibular intercanine and intermolar widths were measured using each company’s software grid feature, as previously described.^14^ The intercanine width was measured from right to left canine cusp tips and rounded to the closest millimeter. The intermolar width was measured from the mesial buccal cusp tips of the first molars from right to left. Differences between final and initial measurements indicated expansion (positive values) or constriction (negative values).

All measurements were conducted by a single investigator (V.T.). To ensure intraoperator reliability, 30% of the sample was reassessed for canine and molar classification, and intercanine and intermolar widths, with a three-week interval between assessments.

### STATISTICAL ANALYSIS

Statistical analysis was performed using SPSS software (SPSS 22.0 Inc., Chicago, IL, USA). Descriptive statistics were employed alongside statistical tests to analyze the data. For paired comparison of variables within each patient’s VTS, the repeated measures ANOVA test was applied to the quantitative variables: number of aligners, number of attachments, amount of IPR per arch, final mandibular intercanine width, and final mandibular intermolar width.

Categorical variables, specifically the final canine and molar relationships, were analyzed using the chi-square test. 

For the extrusion/intrusion of maxillary central incisors, absolute values were used in the repeated measures ANOVA test. To determine agreement, vertical movements were categorized as “E” for extrusion, “I” for intrusion, and “NC” for no change, and analyzed using the chi-square test. This same approach was utilized for the assessment of expansion or constriction of the intercanine and intermolar widths, with “E” for expansion, “C” for constriction, and “NC” for no change. 

The p-values of ≤0.05 were considered statistically significant. Since this was a pilot study, a *post-hoc* power analysis was conducted after analyzing the results.

## RESULTS

The intraclass correlation coefficient showed high reliability for intercanine and intermolar width measurements, with coefficients of 0.972 and 0.949, respectively. Additionally, Kappa agreement values were 0.842 for final canine classification and 1.00 for final molar classification, with both p< 0.001, indicating excellent agreement.

This pilot study aimed to assess various parameters among different clear aligner companies. The raw data are presented in Supplementary Table 1, with mean values and standard deviations shown in Table 3, where statistically significant differences are highlighted. The repeated measures ANOVA test, corrected using the Greenhouse-Geisser method due to sphericity violations, identified significant differences in the number of aligners (p = 0.003), and attachments (p < 0.001). 

**Table 3:** Descriptive statistics and ANOVA results for clear aligner parameters by company.

Clear aligner company			Number of aligners			Number of attachments			Upper IPR (mm)			Lower IPR (mm)		
	n	Mean	SD	p-value	Mean	SD	p-value	Mean	SD	p-value	Mean	SD	p-value	
Invisalign^®^	10	26.8	10.87	0.003*	15.8	2.70	0.001*	0.21	0.66	0.211	0.78	0.92	0.529	
ClearCorrect^®^	10	19.7	9.07		9	2.91		0.50	0.92		0.59	0.88		
3M™ Clarity™	10	32.7	16.85		17.8	5.33		0.00	0.00		0.58	0.90		
Spark™	10	23.7	10.82		14.3	3.40		0.10	0.32		0.37	0.70		
Clear aligner company			Extrusion/E (+) / Intrusion/I (-) of U1s					Class I canine			Class I molar			
	n	Mean	SD	p-value	E	I	NC	p-value	Yes	No	p-value	Yes	No	p-value
Invisalign^®^	10	1.11	1.14	0.250	6	3	1	0.203	6	4	0.013*	9	1	0.473
ClearCorrect^®^	10	0.66	0.76		6	4	0		1	9		10	0	
3M™ Clarity™	10	0.90	1.04		6	4	0		7	3		8	2	
Spark™	10	0.85	1.03		2	8	0		2	8		8	2	
Clear aligner company			Final ICW			Expansion/E (+) / Constriction/C (-) of ICW								
	n	Mean	SD	p-value	Mean	SD	p-value	E	C	NC	p-value			
Invisalign^®^	10	27.60	0.73	0.367	2.4	2.22	0.565	2	7	1	0.884			
ClearCorrect^®^	10	28.40	0.62		2.2	1.99		4	4	2				
3M™ Clarity™	10	27.90	0.74		2.7	2.26		3	6	1				
Spark™	10	28.10	0.64		2.7	1.95		4	5	1				
Clear aligner company			Final IMW			Expansion/E (+) / Constriction/C (-) of IMW								
	n	Mean	SD	p-value	Mean	SD	p-value	E	C	NC	p-value			
Invisalign^®^	10	44.70	2.79	0.068	2.0	1.33	0.470	5	4	1	0.225			
ClearCorrect^®^	10	45.50	3.21		2.0	1.97		6	4	0				
3M™ Clarity™	10	45.80	2.44		2.0	0.88		6	4	0				
Spark™	10	44.20	1.62		2.0	1.03		1	8	1				

*indicates statistical difference based on p-values ≤ 0.05; IPR - interproximal reduction.

NC = no change; ICW = intercanine width; IMW = intermolar width.

Table 4 presents the repeated measures ANOVA pairwise comparison for the variables: number of aligners, number of attachments, and final intermolar width. ClearCorrect^®^ had the lowest average number of aligners (19.7) and attachments (9 per patient), while 3M™ Clarity™ had the highest averages, with 32.7 aligners and 17.8 attachments per patient.

**Table 4:** Pairwise comparison of aligner treatment parameters by company using repeated measures ANOVA.

Pairwise comparison	Number of aligners	Number of attachments	Final IMW
	p-value	p-value	p-value
Invisalign^®^ X ClearCorrect^®^	0.050*	< 0.001*	0.317
Invisalign^®^ X 3M™ Clarity™	0.621	1.000	0.019*
Invisalign^®^ X Spark™	0.199	1.000	1.000
ClearCorrect^®^ X 3M™ Clarity™	0.023*	0.002*	1.000
ClearCorrect^®^ X Spark™	0.239	0.005*	0.912
3M™ Clarity™ X Spark™	0.060	0.025*	0.134

* indicates statistical difference base on p-values ≤ 0.05; IMW = intermolar width

The chi-square revealed a significant difference in the predicted final canine relationship (p = 0.013). Specifically, Invisalign^®^ and 3M™ Clarity™ presented a bilateral canine Class I relationship in 65% of the setups (6 and 7 cases, respectively). In contrast, ClearCorrect^®^ and Spark™ only reached this outcome in 15% of cases (1 and 2 cases, respectively) (Table 3).

No statistically significant differences were observed for other variables. A *post-hoc* power analysis conducted using G*Power (v 3.1) for the variable “number of aligners”, using an alpha level of 0.05 and a sample size of 10 showed an effect size of 0.14 and a statistical power of 0.99, considering the observed standard deviation and correlation among the repeated measures.^15^ The sample size calculation allowed a minimum difference of 10 aligners to be detected, considering the mean standard deviation.

## DISCUSSION

The clear aligner market is evolving, with new companies offering diverse approaches to aligner therapy. Each company differentiates itself through unique material compositions, thickness, gingival trim designs, auxiliary tools, and orthodontic movement strategies. However, approximately 90% of publications focus solely on Invisalign^®^,^7^ leaving a significant knowledge gap regarding other brands. This study, to our knowledge was the first of its kind, aimed to fill this gap by comparing the VTS across different clear aligner companies for the same patient.

To effectively compare the VTS, specific variables critical for assessing treatment efficacy were selected. The total number of aligners was chosen because it correlates with the overall treatment duration. This metric is particularly relevant as some companies claim enhanced efficiency by prescribing fewer aligners. In our study, ClearCorrect^®^ showed the lowest average number of aligners per patient, while 3M™ Clarity™ exhibited the highest average. Exploratory data analysis highlighted that 3M™ Clarity™ tends to prescribe more aligners particularly in cases involving extractions, where more stages are often required to achieve controlled tooth movements and closure of extraction spaces. Additionally, 3M™ Clarity™ employs a unique approach by offering two distinct plastic thicknesses - “Flex” (0.625 mm) and “Force” (0.75 mm) - which might contribute to the higher number of aligners prescribed. Specifically, “Flex” plastic is utilized for movements involving rotation and proclination, whereas the “Force” is employed for corrections such as expansion, torque, bodily movement, and segmental intrusion. This staged approach, leveraging different materials for specific treatment phases, increases the number of aligners required and likely extends treatment duration.^11^ ^,^ ^16^ It could be argued that implementing a more controlled tooth movement regimen could potentially yield an improved outcome and reduce the necessity for refinements, possibly reducing overall treatment time. However, some studies have shown that varying thickness do not play a significant role in the forces and moments generated by clear aligners and that the mechanical behavior of clear aligners with thickness of 0.625 mm and 0.75 mm is similar concerning rotational moments, labio-lingual tipping, and bodily movement.^17^ ^,^ ^18^ Consequently, 3M’s claim that their different material thicknesses perform better for different types of movements does not seem to be supported by the available scientific evidence. Although Spark™ also offers two types of plastic - TruGen™ and TruGen XR™ - the VTS does not combine both plastics within a single case.^12^ Therefore, this study only reflects the use of TruGen™ material. These findings underscore the importance of considering the relationship between the number of aligners, treatment complexity, and clinical objectives when selecting an aligner system. In cases that require precise, incremental adjustments - such as those involving complex tooth movements or extraction spaces - a system like 3M™ Clarity™ may offer advantages due to its staged approach with differentiated plastic thickness. Conversely, ClearCorrect’s strategy of prescribing fewer aligners may be suitable for simpler cases or patients seeking a shorter overall treatment duration. However, this approach may compromise the precision required for complex movements, potentially necessitating more refinements.

The number of attachments was chosen as a variable due to companies’ claims of achieving efficient tooth movement with fewer attachments. Attachments are crucial in clear aligners therapy as they improve retention and enhance control and predictability of tooth movement. However, despite their functional importance, patients often dislike them due to aesthetic concerns and discomfort.^19^

The effectiveness of force transmission in aligners is influenced by both the attachment’s design and the trim of the aligners are intimately related. Studies show that a scalloped trim at the gingival level - the default standard trim employed by Invisalign^®^ and Spark™ - is less effective in transferring forces to the teeth.^20^ ^,^ ^21^ This reduced efficacy is attributed to higher flexibility and weakening of the material at the margins. Elshazly et al^21^found that a straight trim design, extending 2 mm beyond the gingival margin, as employed by ClearCorrect^®^, increases stiffness, retention, and gingival adaptation, leading to better stress distribution and control of tooth movement. By applying more force near the gingival area, closer to the center of resistance, the probability of bodily movement increases.^21^ ClearCorrect^®^ claims to reduce the number of attachments by using a higher trim, positioned 2 mm above the gingival margin.^10^ In our study, ClearCorrect^®^ prescribed fewer attachments than other companies, aligning with their marketing claims. This is clinically relevant for patients who prioritize aesthetics, as fewer attachments may provide a more discrete look of the aligners. In contrast, 3M™ Clarity™, which uses a flat trim at the gingival line, prescribed the highest number of attachments, even higher that Invisalign^®^ and Spark™, which have scalloped trim lines. This finding suggests that 3M™ Clarity™ aligners might not benefit from the mechanical advantages of a higher trim line.

The amount of IPR per arch was selected as a variable since it is a common clinical procedure used to manage crowding, address Bolton discrepancy, and improve overjet and posterior interdigitation.^22^ ^,^ ^23^ In the context of clear aligner treatment, IPR is also employed to resolve areas of collision between teeth that could impede proper tooth movement. Because intraoral scans do not accurately capture interproximal areas, each clear aligner software uses mathematical algorithms in a process known as “shape assumption” which estimates and reconstructs these regions based on available data. The process of “shape assumptions” can vary between companies, influencing the determination of collision area and the amount of IPR prescribed.^24^ ^,^ ^25^ Despite these variations in software algorithms, our study found no statistical difference in the amount of IPR prescribed per arch across different companies. Furthermore, exploratory evaluation of the VTS revealed that there was no pattern in the location of the prescribed IPR, suggesting that the clinical characteristics of each case were not considered. The primary reasons determining the necessity and location of IPR should be clinical factors, such as crowding, Bolton discrepancy, enamel availability, and the presence of black gingival triangles, rather than tooth collision areas determined by the software.^22^ ^,^ ^23^ ^,^ ^26^ Indiscriminate IPR can introduce iatrogenic tooth size discrepancy and have negative effects on canine and molar relationships, overjet and overbite, and can reduce enamel thickness, potentially leading to dentinal exposure and tooth sensitivity.^22^ ^,^ ^26^ Therefore, clinicians should rely on critical thinking and individualized clinical needs rather than depending solely on software recommendations when determining the use and location of IPR in treatment planning.

The vertical movement of the maxillary central incisors was selected as a variable since their final position is crucial for smile aesthetics. Our study revealed no statistical difference in this movement among the companies. Data for this vertical movement was obtained from the tooth movement tables provided by each company’s software. However, recent studies indicate that each software calculates tooth movement differently due to the “shape assumption” and the varying methods used for the determination of the center of rotation of teeth.^24^ ^,^ ^25^ Therefore, a direct comparison of the tooth movement table values between companies might be potentially inaccurate. Clinicians should interpret tooth movement data with caution and rely on clinical judgment to assess whether the projected movements align with desired aesthetic outcomes.

The final predicted canine and molar relationships were selected as metrics to assess whether companies plan for an ideal final occlusion. Our study found that while most setups projected a final bilateral Class I molar occlusion, 60.9% did not exhibit a final Class I canine occlusion (Table 3). Notably, Invisalign^®^ and 3M™ Clarity™ projected a bilateral canine Class I relationship in 6 and 7 cases, respectively, demonstrating a relatively higher success rate in achieving ideal canine occlusion. In contrast, ClearCorrect^®^ and Spark™ achieved this in only 1 and 2 cases, respectively. This discrepancy highlights the potential need for clinicians to adjust these virtual treatment setups to improve the final occlusion, possibly affecting other study variables. As the virtual treatment planning process begins with positioning the teeth in the final desired position, from which the software calculates tooth movement, it raises the question of whether ClearCorrect’s approach of prescribing fewer aligners and attachments correlates with their suboptimal occlusal outcomes.

The final intercanine and intermolar widths were chosen as variables to evaluate differences in expansion, constriction, and final arch form across companies. Alongside IPR and incisor proclination, expansion is commonly used to relieve crowding.^14^ ^,^ ^27^ Ideally, the intercanine width should not be expanded, and the intermolar width expansion should be limited to 2 mm per quadrant, as overexpansion can contribute to instability and relapse.^14^ ^,^ ^28^ Our findings indicate that all companies achieved similar final intercanine and intermolar widths, with an average change in intercanine width ranging from 2.2 to 2.7 mm, and intermolar width of 2.0 mm. This increase in intercanine width may compromise long-term stability of the treatment outcome.^28^ Although 3M™ Clarity™ exhibited greater intermolar width change compared to Invisalign^®^, the difference was not statistically significant.

This research acknowledges several limitations to aid the improvement of future studies comparing VTS among clear aligner companies. This research was a pilot study on a convenience sample, so it lacked randomization and comprised heterogenous cases. To compensate for this limitation, the cases were paired for comparative analysis, although achieving a perfect match was impractical due to variations in clinical preferences and case prescriptions across companies. Although a post hoc power analysis was conducted and suggested a high level of power, this method undermines the premise of randomness, as the calculation is conducted on data that has already been analyzed and reported.^29^ Therefore, it is advisable to analyze the results of this research with caution. Another limitation pertains to potential variability introduced by laboratory technicians, although efforts were made to rely on each software’s algorithms by avoiding written instructions. Lastly, it is well-documented in the literature that virtual treatment setups are predictive models and do not accurately represent actual treatment outcomes.^27^ ^,^ ^30^ To achieve an ideal occlusion at the end of treatment, refinement sets may be necessary, which might impact the presented results.

Our findings suggest that the unique characteristics of each aligner company leads to distinct approaches in treating the same patient, highlighting both areas of discrepancy and consistency in the virtual treatment setups. Based on our study, if Invisalign^®^ is considered the gold standard for comparison, there are no clinical differences between them and Spark™, save for a possible deficiency of the latter in planning for a final Class I canine relationship. ClearCorrect^®^ would allow for faster treatments, with fewer aligners and attachments, but also with a deficiency in achieving final Class I canine relationship. And finally, 3M™ Clarity™ would lead to longer treatments, with more aligners and higher number of attachments, achieving a satisfactory final outcome.

## CONCLUSIONS

The virtual treatment setups for the same patient vary significantly among clear aligner companies in terms of number of aligners, number of attachments, and the predicted final canine relationship. However, no statistical differences were observed for the amount of IPR per arch, the planned extrusive or intrusive movements of maxillary central incisors, the predicted final molar relationship, and the predicted final intercanine and intermolar widths, including the planned expansion or constriction of these widths. 

ClearCorrect^®^ stands out by prescribing the fewest number of aligners and attachments, whereas 3M™ Clarity™ tends to prescribe the highest number for both.

Most virtual treatment setups projected a final bilateral molar Class I relationship, regardless of the company. However, there was a notable deficiency across companies in planning for a final bilateral canine Class I relationship, particularly with ClearCorrect^®^ and Spark™.

## Supplementary table 1: Raw data on virtual treatment setup parameters across clear aligner companies

[Table t1app]

**Table t1app:**

Patient	Clear aligner company	Number of aligners	Number of attachments	Upper IPR	Lower IPR	E(+) / I(-) of U1s	Class I canine	Class I molar	Final ICW	E(+) / C(-) of ICW	Final IMW	E(+) / C(-) of IMW
1	Invisalign®	47	16	0	0	2.35	Yes	No	26	0	40	-4
	ClearCorrect®	31	10	0	0	2.21	No	Yes	28	2	41	-3
	3M™ Clarity™	52	24	0	0.4	0.85	Yes	Yes	28	2	42	-2
	Spark™	40	20	0	0	1.25	No	Yes	30	4	42	-2
2	Invisalign®	35	16	0	0.4	-0.80	Yes	Yes	32	7	46	-3
	ClearCorrect®	30	9	0	0	0.24	No	Yes	32	7	46	-3
	3M™ Clarity™	64	24	0	0	-0.50	Yes	Yes	33	8	47	-2
	Spark™	38	16	0	0	-0.35	No	Yes	30	5	46	-3
3	Invisalign®	18	19	0	1.2	3.80	No	Yes	29	-1	47	1
	ClearCorrect®	10	9	0.9	1.7	1.83	No	Yes	28	-2	49	3
	3M™ Clarity™	20	18	0	1.4	3.55	No	No	28	-2	49	3
	Spark™	19	14	0	0	3.50	No	Yes	29	-1	45	-1
4	Invisalign®	32	16	0	0.6	1.20	Yes	Yes	24	-4	44	-3
	ClearCorrect®	29	14	0	0	0.28	No	Yes	25	-3	46	-1
	3M™ Clarity™	34	23	0	0	1.40	Yes	Yes	24	-4	46	-1
	Spark™	32	15	0	0	-1.45	Yes	Yes	25	-3	45	-2
5	Invisalign®	17	20	0	2.1	-0.40	Yes	Yes	25	-1	43	1
	ClearCorrect®	23	8	0	0.3	-0.47	Yes	Yes	28	2	45	3
	3M™ Clarity™	21	15	0	0	-1.30	No	No	26	0	45	3
	Spark™	15	13	0	0	-0.35	No	No	26	0	41	-1
6	Invisalign®	15	12	2.1	0	0.35	No	Yes	28	-1	49	1
	ClearCorrect®	12	4	1.4	0	-0.04	No	Yes	29	0	50	2
	3M™ Clarity™	19	15	0	0	0.05	Yes	Yes	27	-2	49	1
	Spark™	13	13	0	0.6	-0.15	No	Yes	27	-2	45	-3
7	Invisalign®	15	12	0	0	0.00	No	Yes	29	-1	45	0
	ClearCorrect®	10	7	2.7	0	0.85	No	Yes	29	-1	46	1
	3M™ Clarity™	25	10	0	0	0.45	No	Yes	29	-1	46	1
	Spark™	10	8	1	1	-0.65	No	Yes	29	-1	45	0
8	Invisalign®	36	18	0	1	0.40	No	Yes	29	-1	41	-3
	ClearCorrect®	27	9	0	0.2	0.54	No	Yes	30	0	40	-4
	3M™ Clarity™	51	23	0	0	0.20	Yes	Yes	29	-1	42	-2
	Spark™	32	18	0	0	-0.30	No	No	31	1	43	-1
9	Invisalign®	23	14	0	2.5	0.95	Yes	Yes	27	-4	47	1
	ClearCorrect®	11	7	0	1.3	-0.01	No	Yes	29	-2	48	2
	3M™ Clarity™	17	12	0	2.5	-0.20	Yes	Yes	28	-3	47	1
	Spark™	18	11	0	2.1	-0.20	No	Yes	26	-5	45	-2
10	Invisalign®	30	15	0	0	-0.85	Yes	Yes	27	4	45	3
	ClearCorrect®	14	13	0	2.4	-0.18	No	Yes	26	3	44	2
	3M™ Clarity™	24	14	0	1.5	-0.45	Yes	Yes	27	4	45	3
	Spark™	20	15	0	0	-0.30	Yes	Yes	28	5	45	3

IPR - Interproximal reduction; E(+) / I(-) of U1s - Extrusion or intrusion of maxillary central incisors; ICW - intercanine width; IMW - intermolar width; E(+) / C(-) of ICW/IMW - Expansion or constriction of ICW or IMW.
